# Genotyping and Phylogenetic Analysis of *Yersinia pestis* by MLVA: Insights into the Worldwide Expansion of Central Asia Plague Foci

**DOI:** 10.1371/journal.pone.0006000

**Published:** 2009-06-22

**Authors:** Yanjun Li, Yujun Cui, Yolande Hauck, Mikhail E. Platonov, Erhei Dai, Yajun Song, Zhaobiao Guo, Christine Pourcel, Svetlana V. Dentovskaya, Andrey P. Anisimov, Ruifu Yang, Gilles Vergnaud

**Affiliations:** 1 Laboratory of Analytical Microbiology, State Key Laboratory of Pathogen and Biosecurity, Institute of Microbiology and Epidemiology, Beijing, China; 2 Université Paris-Sud 11, CNRS, UMR8621, Institut de Génétique et Microbiologie, Orsay, France; 3 State Research Center for Applied Microbiology and Biotechnology, Obolensk, Moscow Region, Russia; 4 DGA/D4S - Mission pour la Recherche et l'Innovation Scientifique, Bagneux, France; St. Petersburg Pasteur Institute, Russian Federation

## Abstract

**Background:**

The species *Yersinia pestis* is commonly divided into three classical biovars, Antiqua, Medievalis, and Orientalis, belonging to subspecies *pestis* pathogenic for human and the (atypical) non-human pathogenic biovar Microtus (alias Pestoides) including several non-*pestis* subspecies. Recent progress in molecular typing methods enables large-scale investigations in the population structure of this species. It is now possible to test hypotheses about its evolution which were proposed decades ago. For instance the three classical biovars of different geographical distributions were suggested to originate from Central Asia. Most investigations so far have focused on the typical *pestis* subspecies representatives found outside of China, whereas the understanding of the emergence of this human pathogen requires the investigation of strains belonging to subspecies *pestis* from China and to the Microtus biovar.

**Methodology/Principal Findings:**

Multi-locus VNTR analysis (MLVA) with 25 loci was performed on a collection of *Y. pestis* isolates originating from the majority of the known foci worldwide and including typical rhamnose-negative subspecies *pestis* as well as rhamnose-positive subspecies *pestis* and biovar Microtus. More than 500 isolates from China, the Former Soviet Union (FSU), Mongolia and a number of other foci around the world were characterized and resolved into 350 different genotypes. The data revealed very close relationships existing between some isolates from widely separated foci as well as very high diversity which can conversely be observed between nearby foci.

**Conclusions/Significance:**

The results obtained are in full agreement with the view that the *Y. pestis* subsp. *pestis* pathogenic for humans emerged in the Central Asia region between China, Kazakhstan, Russia and Mongolia, only three clones of which spread out of Central Asia. The relationships among the strains in China, Central Asia and the rest of the world based on the MLVA25 assay provide an unprecedented view on the expansion and microevolution of *Y. pestis*.

## Introduction

Plague, one of the most devastating infections in human history, is a zoonotic infection that spreads to humans from natural rodent reservoirs, commonly via the bite of an infected flea [1]. *Yersinia pestis*, the causative agent of plague, is a multi-host and multi-vector pathogen, involving more than 200 species of wild rodents as host and over 80 species of fleas as vector [2]. Different hosts and vectors have their own specific ecological landscape and different levels of susceptibility to the organism [3]. In addition survival of the bacteria in the soil is likely to contribute to the long-term persistence of *Y. pestis* foci [4], [5]. During its expansion and adaptation to new niches, *Y. pestis* undergoes genetic variations [6], some of which may help overcome natural selective forces. These variations may be used as markers to reconstruct the historical spread of plague.

Because of its importance in human history, many investigations have aimed at deciphering the evolution of this major pathogen. In the decades following its identification by Alexandre Yersin in 1894, biochemical characteristics were identified, allowing the typing of strains. Some of these characteristics could be shown to constitute a strong phylogenetic signal. This led to the well-known classification of the typical human pathogenic *Y. pestis* (subspecies *pestis* in Russian nomenclature) into three biovars, bv. Antiqua, bv. Medievalis and bv. Orientalis [7]. With only very few exceptions [8], [9], the corresponding biochemical behavior of Medievalis or Orientalis isolates (Antiqua simply reflects a more ancestral behavior) was subsequently associated with a unique ancestral mutation event within *Y. pestis* subsp. *pestis* which explains the strong phylogenetic value of this classification. Since the corresponding assay could be routinely and easily run by any microbiology laboratory, typing data from different groups could be merged and compared without the need to exchange live strains. Based upon historical records and known plague foci, Devignat [7] proposed that Justinian plague, which started in the sixth century from Africa was due to the *Y. pestis* subsp. *pestis* biovar still present in some Central African plague foci, which he called Antiqua for this reason. He suggested that the Antiqua natural foci established in Africa were the result of earlier invasions by Aryans coming from Asia, 1000–2000 years B. C. Using the same line of reasoning, Devignat proposed that the biovar present in Central Asia and around the Caspian Sea was responsible for the second pandemic of plague in the Middle Ages. For this reason he called this biovar “Medievalis”. The third pandemic which considerably extended the geographic extension of plague, was in progress when Yersin first isolated the organism and can be unequivocally assigned to a third biovar, which Devignat called “Orientalis” (coming from the Orient, *i.e.* East). In this model, all three biovars originated from Central Asia and China. This is still not a consensus view. Other authors have suggested very early that only “Orientalis” strains could induce plague in humans (reviewed in [7]). More recently some reports suggested that DNA specific for “Orientalis” was present in remnants from all three pandemics (see [10]; this report was subsequently shown to contain significant technological flaws [11]).

The three-biovar classification was initially intended to be applied to rhamnose-negative *Y. pestis* subsp. *pestis* strains. We propose in Table S1 to designate the rhamnose-positive strains, occasionally infecting humans, and with marmots as main host, described mostly in foci A and B in China [12] as a new biovar, bv. Intermedium, to avoid the frequent confusion with bv. Antiqua. Biovar Intermedium strains belong to *Y. pestis* subsp. *pestis* (as confirmed by molecular analysis [12]). Interestingly, a number of other plague foci associated with rhamnose-positive strains have been described in China and the Former Soviet Union (FSU) [2], [13], [14]. A new biovar, Microtus, was previously proposed to include strains isolated from voles as main host (*Microtus brandti* and *Microtus fuscus*) [12], [15], [16] (Table S1). They are usually rhamnose-positive and of low virulence or avirulent for guinea pig [2]. This biovar also includes strains from the FSU, which were formerly classified as Pestoides and more recently as a number of subspecies, called *caucasica*, *altaica*, *ulegeica*, *hissarica*, and *talassica* [17]. Some bv. Microtus strains do not reduce nitrates (Table S1), and are then sometimes called bv. “Medievalis” to reflect this behavior but this is misleading in terms of phylogeny since the causative mutation is different from the one found in typical human pathogenic *Y. pestis* subsp. *pestis* bv. Medievalis [15] (in the rest of this report, we will use the currently commonly accepted naming convention recalled in Table S1 in spite of inconsistencies in the conventional hierarchical order between biovars and subspecies).

Much more discriminatory molecular typing methods have been described in the past few years. Some of these methods can be used in many laboratories to produce data which can be easily merged and compared without the need to exchange live strains, or other biological material. As a result, it is now possible to re-examine the founding hypotheses and the classifications underlying them. These methods include single nucleotide polymorphism (SNP) analysis [9], [12], regions of deletion analysis, [18], [19], whole-genome sequencing [20], clustered regularly interspaced short palindromic repeats (CRISPRs) analysis [17], [21], [22] and Multiple Loci Variable number of tandem repeats (VNTR) Analysis (MLVA) [8], [9]. MLVA was shown in a number of bacterial species to be capable of distinguishing closely related strains and successfully classifying more distant relationships [23] in spite of the intrinsic homoplasy of VNTR loci, at least when using the appropriate loci. Pourcel *et al*. [8] and Achtman *et al*. [9] examined strains of *Y. pestis* by using 25 and 42 VNTR loci, respectively.

Until now the available MLVA data of *Y. pestis* is derived from a small number of strains, especially those from western countries which essentially represent natural foci resulting from the third pandemic, a few natural foci from Africa, Iran, Turkey, and a few strains from some of the natural foci in Asia [6], [8], [9], [24]–[27]. Little is known for instance about the rhamnose-positive bv. Intermedium strains [12].

In this study, we used 25 VNTR markers evaluated by Pourcel *et al*. [8] to analyze 383 strains of *Y. pestis* from different hosts in different natural plague foci around China as well as 38 representative strains from the FSU and Mongolia. We then compared the results with previously published data from 180 isolates [8].

## Materials and Methods

### Strains and DNA

Three hundred and eighty-three *Y. pestis* strains from eighteen different natural plague foci in China were identified as *Y. pestis* by bacteriophage lysis using phage YP-ChinaPhage1 (unpublished assay, ongoing phage sequencing project). Thirty-eight strains from 15 foci in the FSU and Mongolia, which were isolated from different hosts and plague patients, were selected to represent the diversity of this bacterium in Central Asia (Table S2). All the strains in China were previously characterized by biochemical assays and by different genotyping methods, including different region (DFR) and pseudogene profiling [12], [28]–[30]. The bacteria were cultivated in LB broth at 28°C for 24 hours and the chromosomal DNA was extracted using conventional SDS lysis and phenol-chloroform extraction method.

One *Yersinia pseudotuberculosis* isolate from China which was initially misidentified as *Y. pestis* using the phage typing assay was included in the analysis.

### Natural plague foci nomenclature

Chinese natural plague foci are lettered (A to O) e.g. “focus B” [18] and some foci are subdivided using numbers, e.g. focus B1 to B4; natural plague foci from the FSU are numbered e.g. “focus 34” [2]; natural plague foci of Mongolia are named by “focus + the first two letters of the province name of origin”, like “focus BP” for Bayanölgie Province (aymag) of Mongolia. The geographic position and background data for the natural plague foci were documented in previous reports [2], [8], [18], [31].

### MLVA markers and PCR amplification

The 25 VNTR markers ms01, ms04 (M58), ms05 (M59), ms06, ms07 (M37), ms09, ms15, ms20 (M51), ms21, ms35, ms38, ms40, ms41, ms44, ms45 (M42), ms46, ms51, ms54 (M72), ms56, ms62 (M34), ms69, ms70, ms71, ms73 and ms74 were previously described [8] (the names indicated in brackets refer to the aliases used by Klevytska *et al*. [27]). The main characteristics of the 25 loci are listed in Table 1 including the diversity index of each locus.

**Table 1:** Main characteristics of the 25 loci.

Locus^a^	Number of alleles	Allele size range(units)	Polymorphism index
YPO0120ms01_18bp_228bp_8U	6	5–10	44.9
YPO1290ms04_17bp_230bp_8U	9	2–10	70.4
YPO1935ms05_17bp_291bp_11U	8	2–4, 6, 8–11	52.7
YPO2769ms06_60bp_606bp_8U	14	4–15, 17, 25	77.1
YPO2916ms07_10bp_184bp_9U	9	3–11	66.5
YPO3057ms09_18bp_682bp_33U	21	5, 7–11, 21–24, 26, 32–40	87.2
YPO0559ms15_15bp_237bp_10U	2	1–2	8.5
YPO1814ms20_15bp_253bp_9U	4	5, 8–10	47.7
YPO1895ms21_18bp_278bp_9U	6	4, 9–13	11.3
YPO4042ms35_15bp_204bp_8U	5	3, 7–10	55.0
YPO4425ms38_16bp_233bp_8U	6	5–8, 10, 11	58.4
YPO0581ms40_17bp_214bp_7U	5	1, 7–9, 11	53.7
YPO0718ms41_17bp_217bp_7U	4	2, 5–7	50.8
YPO1018ms44_17bp_233bp_7U	4	2, 5–7	5.1
YPO1108ms45_12bp_161bp_7U	5	2–6	21.3
YPO1335ms46_7bp_112bp_5U	19	4–21, 24	88.5
YPO2058ms51_21bp_207bp_2U	3	2–4	22.6
YPO2612ms54_22bp_281bp_7U	5	4–8	46.5
YPO3060ms56_16bp_220bp_7U	7	2–8	72.5
YPO4280ms62_9bp_124bp_7U	23	3–25	92.6
YPO1118ms69_16bp_179bp_6U	4	3, 5–7	44.6
YPO1580ms70_9bp_146bp_6U	10	2–9, 11, 12	76.8
YPO1925ms71_14bp_171bp_6U	6	3–8	43.8
YPO3236ms73_18bp_225bp_6U	4	3–6	44.1
YPO3245ms74_15bp_195bp_6U	6	2, 5–9	49.0

a the indicated locus name includes the full allele coding convention (including the repeat unit number in the first *Y. pestis* genome sequence published, from strain CO92).

The primers used for amplification of these 25 minisatellite loci were the same as those reported initially [32] except for ms46 and ms62, for which new primer sets were designed in order to yield smaller PCR products allowing better resolution on agarose gels (YPO1335ms46-L: TAGACTTTACTCGCGGCTAGC, YPO1335ms46-R: ATAATGTTAGTGGCGAGTCGTC; YPO4280ms62-L: GCGGGTTGACGCTGTTGAGCAAC, YPO4280ms62-R: TTGTTCTTGAGCCGCTACCGGGAT). Two errors in previous reports [8], [32] were identified and corrected. The first error is that ms46 was previously identified as a 17 bp repeat unit VNTR with intermediate size alleles whereas it is a 7 bp repeat unit (with no intermediate size alleles). The new coding convention for ms46 is summarized as YPO1335ms46_7bp_112bp_5U, meaning that using the new primer set, a 112 bp PCR product is expected in the first published *Y. pestis* sequence (strain CO92, accession number AL590842) and by convention this size corresponds to 5 repeat units. The second error is that ms51 is a 21 bp (instead of 18 bp) repeat unit VNTR. The new coding convention is summarized as YPO2058ms51_21bp_207bp_2U (Table 1). A volume of 30 µl PCR reaction mixture contained 10 ng of DNA template, 0.5 µM of each primer, 1 unit of Taq DNA polymerase, 200 µM of dNTPs, and 10×PCR buffer (500 mM KCl, 100 mM Tris HCl (pH 8.3) 25 mM MgCl_2_). The amplification was carried out in a DNA thermocycler (MJ Research PTC-225) with denaturation at 95°C for 5 minutes, followed by 30 cycles of denaturation at 95°C for 40 seconds, annealing at 58°C for 40 seconds, elongation at 72°C for 1 minute as previously described [8]. A final 5-minutes elongation at 72°C was performed after the last cycle to ensure complete extension of the amplicons. Six microliters of the PCR products were run on standard 3% agarose gel (Qbiogen) in 0.5×TBE buffer at a voltage of 8–10 V/cm. Gel lengths of 10 to 40 cm were used according to PCR product size and motif length. Gels were stained with ethidium bromide and visualized under UV light. A strain which alleles have been precisely measured either by re-sequencing or by direct comparison with a sequenced reference strains was used as reference (within this project, DNA from Microtus strain 91001 alias L1970003 [15] or the vaccine EV76 strain were used for this purpose). The DNA size marker (20 bp or 100 bp ladder) and amplicons from reference strain were run as controls for alleles size estimation as described previously [33].

### Data merging with previously published data

Published data [8] was merged with the present data set after correcting ms46 and ms51 data as explained. Ms46 and ms62 data were rechecked by retyping one strain from each genotype as described [34].

### Data analysis

Gel images were analyzed using the BioNumerics software package version 6.0 (Applied-Maths, Sint-Martens-Latem, Belgium) as described [8] or most often by visual inspection of the gel images. The number of motifs in each allele was deduced from the amplicon size using the previously published allele calling convention except for ms46, ms62 and ms51 (Table 1). The MLVA profile at the 25 loci was deduced for the sequenced *Y. pestis* strains using the tools available from http://minisatellites.u-psud.fr/. Data produced by agarose gel electrophoresis and visual image analysis, by capillary electrophoresis machines or deduced *in silico* from genome sequence data were imported into BioNumerics by creating a virtual gel image. Gel image data were converted into character data sets. Clustering analysis was done using the categorical coefficient and the Neighbor-Joining method. The tree was rooted using a *Y. pseudotuberculosis* isolate as outgroup.

## Results and Discussion

### MLVA genotyping

The 25 loci could be amplified in most isolates except for YPO1108ms45 and YPO1118ms69. Twenty-nine and 24 isolates failed to yield bands for loci YPO1108ms45 and YPO1118ms69, respectively. These two loci fall within the previously described different region 09 (DFR09) [35].

**Figure 1:**
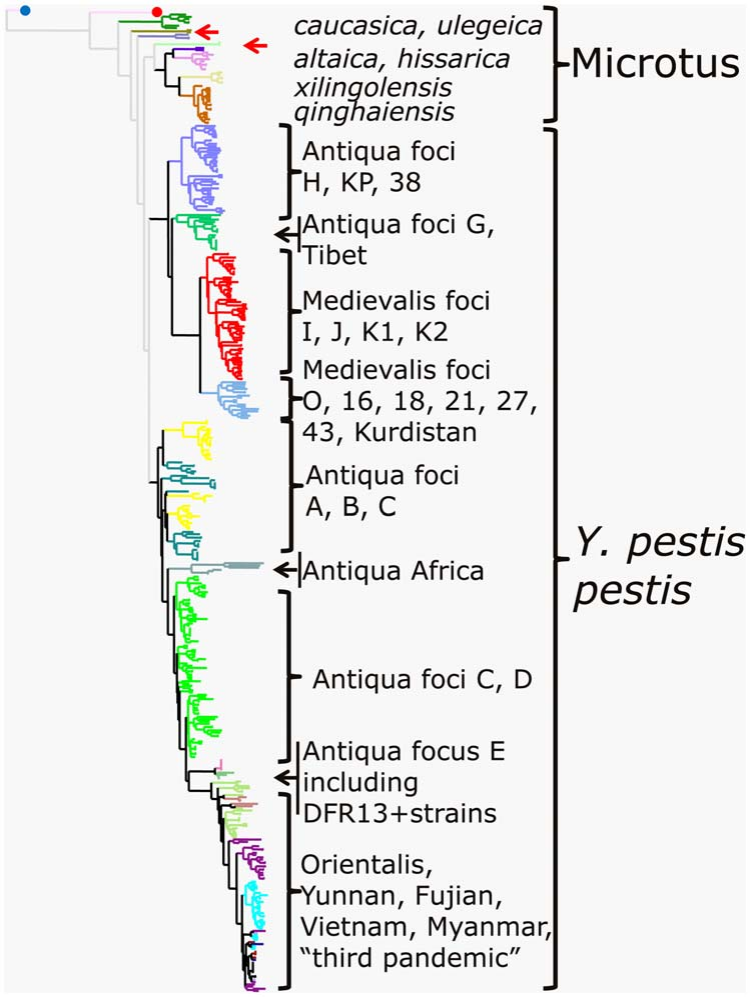
Dendrogram based on the 25 VNTR loci. Clustering analysis was done using the categorical distance coefficient and the Neighbor-Joining clustering method. The *Y. pseudotuberculosis* representative (blue dot) was chosen as outgroup to root the tree. Red dot, Angola isolate. Red arrows, *Y. pestis* subsp. *pestis* isolates with exceptional genotypes. The branches color code is as indicated in legends from Figure 2 and Figures S1, S2, S3.

**Figure 2:**
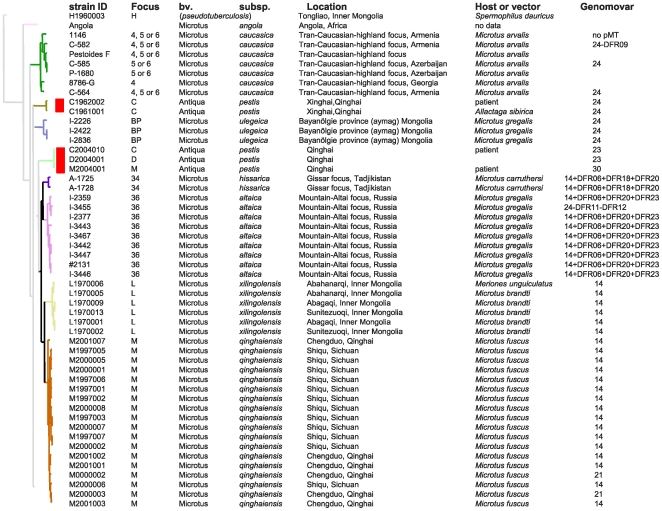
The *Y. pestis* bv. Microtus isolates, dendrogram based on the 25 VNTR loci. From left to right, the columns designate the strain Id, focus of origin, biovar (bv.), subspecies (subsp.), geographic origin (location), host or vector, genomovar based on DFR analysis [28]. The biovar or subspecies designation follows current usage, with inconsistencies in terms of a future nomenclature since “biovar Microtus” contains a number of “subspecies”. The ‘genomovar+DFRX’ and ‘genomovar-DFRX’ respectively indicates that the strain is similar to this genomovar except for DFRX which was present or absent. The branches color code reflects the focus of origin. Five atypical isolates corresponding to essentially two strains fall into two very long and loosely connected branches (red rectangles). At least three were derived from patients.

The data was merged with typing data deduced from sequenced genomes and with published data using the same MLVA assay [8]. A *Y. pseudotuberculosis* isolate from focus H of China which was originally misidentified as *Y. pestis* by the phage lysis identification procedure, was used as an outgroup to root the tree. Three hundred and fifty genotypes were distinguished. Figure 1 provides an overview of the clustering achieved. The bv. Microtus isolates are grouped. The Angola strain is suggested to represent the most ancestral branch. The *caucasica* subgroup (including the sequenced strain “Pestoides F” [36]) is suggested to be in the next most ancestral position (Figure 2) in agreement with previous biochemical investigations [2].

**Figure 3:**
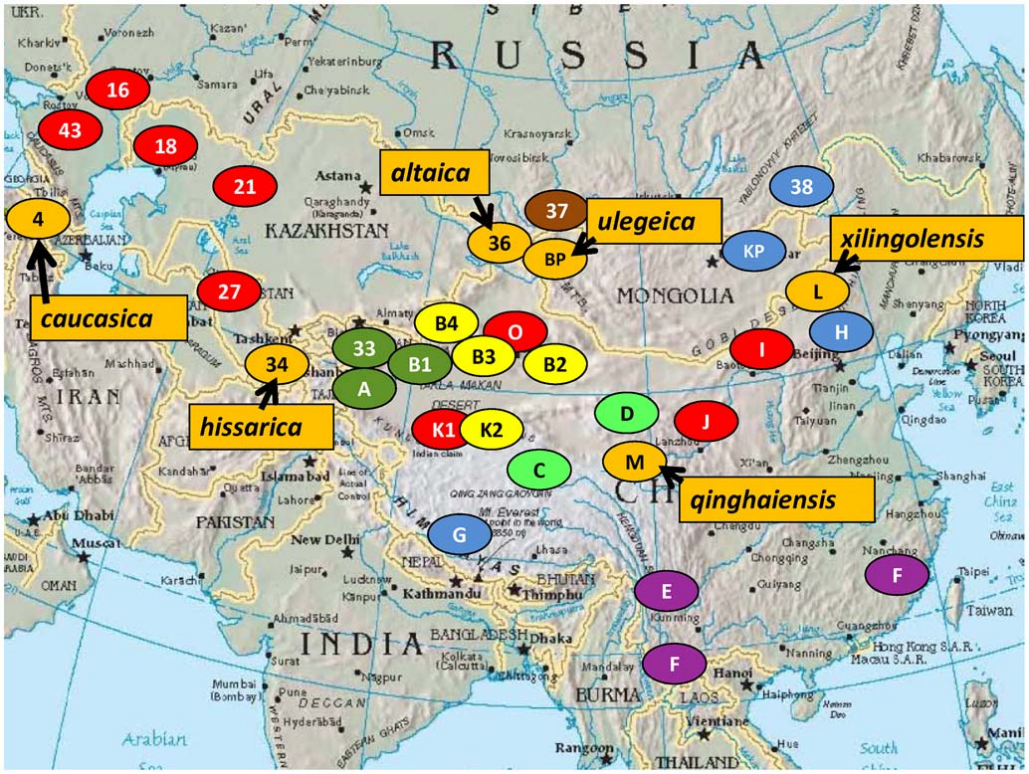
The close relationship among the foci in China and Central Asia. The color code reflects some significant genetic relationships as indicated by MLVA clustering. Orange, the bv. Microtus investigated here, including subspecies *caucasica* (4), *ulegeica* (BP), and the most closely related *hissarica* (34) *altaica* (36), *xilingolensis* (L) and *qinghaiensis* (M). Red foci, *Y. pestis* subsp. *pestis* biovar Medievalis. Purple foci, bv. Orientalis foci. Other colors, different varieties of *Y. pestis* subsp. *pestis* bv. Intermedium and Antiqua strains. The more detailed composition of each focus is presented in Figures 2 and Figures S1, S2, S3.

There are two bv. Microtus-related foci in China, focus L and focus M (Table S1 and Figure 3) corresponding to subspecies *xilingolensis* and *qinghaiensis* respectively [17]. The isolates from these foci cluster with the *hissarica* and *altaica* subspecies from focus 34 and 36 respectively (Figure 2). These four subspecies are suggested to be the most closely related to *Y. pestis* subsp. *pestis* (Figure 1). Subspecies *hissarica* and *altaica* were previously and independently suggested to be closely related to *Y. pestis* subsp. *pestis* by biochemical analysis (reviewed by [2]). Although the L and M foci are geographically distant, the phenotypic features of the *Y. pestis* isolates from these foci were almost identical. Many methods used in recent years failed to differentiate the strains in the two foci until the report of two spacers difference in CRISPR locus YPc [17]. In the present study, the 41 isolates can be well differentiated into their corresponding foci (Figure 2).

Five isolates fall into two very distinct branches (red arrows and red rectangles in Figures 1 and 2 respectively). One branch corresponds to rhamnose-negative strains. Three of these five isolates were isolated from patients (Figure 2). Three originate from the C focus, one from the D, and one from the M focus. In this last case, the patient had recently being travelling back from the C focus.

Except for these 5 exceptions, the *Y. pestis* subsp. *pestis* isolates (main subspecies) fall into two main clusters (Figure 1). The first main cluster contains the bv. Medievalis isolates, and bv. Antiqua isolates from Mongolia, North-East China and Tibet (foci KP, H and G respectively). The sequenced strain Nepal 516 belongs to this last Tibet subcluster as could be expected (green “G” clade, Figure S1). Two main branches are apparent within the bv. Medievalis group. Isolates from the K1, J and I foci are grouped in three subclusters corresponding to their geographic origin. In contrast, the second bv. Medievalis branch contains isolates from a wide range of foci (Figure 1), including focus O in China, as well as the Kurdistan focus explored by Baltazard and colleagues in Iran [31] and other foci located in between (foci 16, 18, 21, 27, 43; [2]). The sequenced strain KIM falls within this group. This result is consistent with that of DFR analysis. Isolates in focus O and the sequenced strain KIM shared identical DFR profiles and belong to genomovar 15 [28]. This observation indicates that strains from focus O from China are closer to the bv. Medievalis strains found in the Central Asian Desert plague foci than from the other Chinese bv. Medievalis foci I, J, K1, and H.

The second main cluster contains bv. Intermedium and Antiqua isolates from foci A, B, C, D together with the whole bv. Orientalis group. This cluster also contains the African bv. Antiqua strains (from the Albert and Edouard lakes in Uganda and Democratic Republic of Congo).

The global organization for *Y. pestis* subsp. *pestis* suggested by MLVA clustering is in agreement with previous investigations. SNP analysis previously identified two different molecular groups of bv. Antiqua strains representing two evolutionary lineages of *Y. pestis* (1.ANT and 2.ANT), associated respectively with the bv. Orientalis and bv. Medievalis branches [9]. Lineage 1.ANT was represented in these earlier investigations by African bv. Antiqua isolates. Similarly whole genome sequence comparison as well as CRISPR analysis has identified two clusters of bv. Antiqua [20], [22]. These two branches were classified as Asian and African but the present study clearly illustrates that both bv. Antiqua clusters are largely represented in China.

In a previous report, 909 Chinese isolates were grouped into 32 genomovars based upon presence-absence of 23 DFRs [28]. Due to the limited resolving power of DFR analysis some strains isolated from different foci were indistinguishable. For instance, most strains from foci G and H, foci K1 and I, and foci L and M shared respectively genomovar 10, genomovar 11 and genomovar 14 (Figure 2 and Figure S1). This suggested a very close relationship between the strains in the corresponding foci. In the present study, these strains could be easily differentiated according to their focus of origin (Figures 2 and 3; Figure S1). Therefore, VNTR analysis with the 25 loci provides much finer resolution compared with DFR analysis while the clustering it achieves is in agreement with the geographic origin of the isolates and the overall DFR results.

### Relationships between strains in China and Central Asia

Three foci in the Xinjiang province of China, foci A, B (including subfoci B1–B4) and O are geographically linked to the plague foci of Central Asia (Figure 3). The Central-Asian-Desert plague foci stretch eastward directly to China, and join with the newly identified focus O, the *Rhombomys opimus* (great gerbil) plague focus of the Junggar Basin of Xinjiang [37]–[39]. The high similarity seen by VNTR analysis of 25 loci between focus O and foci 16, 18, 21, 27, 43 and Kurdistan suggests that the bv. Medievalis isolates from focus O has a close relationship to the Medievalis isolates from the Central-Asian-Desert plague foci (Figure 3).

The 6 isolates from mesofocus (subfocus) 37 (Tuva focus in Russia, brown colored focus, Figure 3) presented close relationships with isolates from foci A, B (and a few C) in Xinjiang province of China (Figure S2, and yellow and dark green colored foci in Figure 3). In the absence of geographical barriers and given the relatively short distances involved (Figure 3), the spread or exchange of *Y. pestis* isolates between these areas is not unlikely. Similarly focus 33 (Aksai focus in Kirghizia) is adjacent to foci A and B1 in Xinjiang province of China and the similarity among the isolates in these 3 foci is not surprising.

The bv. Antiqua group predominantly associated with isolates from the H focus in Song-Liao Plain of China also contains the isolates from focus KP (Khentei Province in Mongolia) and four isolates from focus 38 (Trans-Baikal focus in Russia; blue foci in Figure 3). Genetically closely related bv. Antiqua and bv. Medievalis strains have been isolated from the H focus. It is tempting to speculate that this focus may represent the origin of emergence of bv. Medievalis.

A homogeneous group of bv. Microtus strains as assigned here by MLVA, or previously by DFR (genomovar 14 or minor variants) and CRISPR analysis, is consistently found in foci L (Microtus/*xilingolensis*), M (Microtus/*qinghaiensis*), as well as focus 34 (subspecies *hissarica*, Gissar focus, Tadjikistan) and focus 36 (subspecies *altaica*, Mountain-Altai focus in Russia). Microtus/*angola*, Microtus/*caucasica* (focus 4, 5, 6) and Microtus/*ulegeica* (focus BP) are more distantly related. MLVA analysis, in agreement with biochemical data [2] and DFR analysis suggests that the first two subspecies represent more ancestral branches. It is tempting to speculate that the wide bv. Microtus geographic distribution across Central Asia, China, Mongolia, could have predated the emergence of *Y. pestis* subsp. *pestis*, pathogenic for a number of mammalian species (including humans) larger than *Microtus*. The ancestral *Y. pestis* subsp. *pestis* would have been rhamnose positive. These observations lead to the following clonal expansion model.

### Proposed *Y. pestis* transmission events accounting for the strain distribution in current natural plague foci

The older, non-human pathogens bv. Microtus spread all over Central Asia, China and Mongolia, in the distant past (which may be more than the current estimates of a maximum time of 20 000 years since *Y. pestis* emerged from *Y. pseudotuberculosis* [40] because this estimate does not take into account the possible survival of *Y. pestis* in the soil). Spreading might have gone from West to East, as suggested by the phylogenetic position of the *caucasica* lineage. The current bv. Microtus foci are remnants of this expansion. In particular, foci L, M, 34 and 36 which although geographically very far apart represent the most homogeneous bv. Microtus subspecies, are suggested by MLVA analysis to be the most closely related to *Y. pestis* subsp. *pestis*. The A-B-C-33 foci located nearby focus 34 are very remarkable in terms of bv. Antiqua strains diversity. For instance, two isolates of focus C and one of focus M (from a patient who previously travelled to focus C), biochemically defined as bv. Antiqua, are pathogenic for humans, but are only loosely connected to the *Y. pestis* subsp. *pestis* main clusters according to MLVA clustering. This region is the most likely candidate for the place of emergence of the *Y. pestis* subsp. *pestis* human pathogen. In particular, this is where rhamnose-positive bv. Intermedium strains are found (Table S1). From there, (rhamnose-negative) bv. Antiqua *Y. pestis* subsp. *pestis* strains would have spread to foci E, G and H. One bv. Antiqua strain gave birth to the plague foci present in Central Africa. High resolution SNP typing and whole genome sequence analysis will be needed to more precisely evaluate the relative age of the Caspian Sea Medievalis and African Antiqua branches. The Orientalis biovar presumably emerged from focus E, in which bv. Antiqua strains harboring DFR13 and showing highly similar MLVA patterns are present (Figure S3).

The spread of bacteria is a dynamic process, and the strains persisting in natural plague foci are not fixed. The observed genetic similarities between the different foci suggest that different patterns of transmission and fixation coexist. In some cases, it is likely that localized spreading occurred. Similar terrain and landscape in adjacent area facilitated the transmission of the isolates via their hosts from one location to the other and gradually gave rise to a present-day focus that is slightly different from the original one. This is a common transmission pattern and the evolution of isolates in foci 33, A, and B1 fits this pattern, as well as the foci C, D, and K2 in China. The strain transmission among foci B2, B3, and B4 in Xinjiang province of China also probably results from localized spreading [41], [42].

The second pattern of spread is point-to-point transmission: bacteria from one focus may be carried to a remote region by various events, such as migration of hosts or transportation of goods, or movement of human beings infected by the pathogen. This mode of spread is particularly seen in the *Y. pestis* subsp. *pestis* pathogenic for humans, which have been very successful in their geographic colonization. Long-distance transmission of isolates could lead to two outcomes 1) the bacteria could not adapt to the local surroundings and will disappear (as illustrated for the first, second and third pandemic which did not leave natural foci in Europe, as well as the third pandemic in Australia); 2) where the ecosystem is similar to the original focus, the bacteria could survive and cause epizootics in local hosts, and hence a natural focus with similar isolates would form. The African Antiqua foci would be an illustration. This pattern may account for the fact that isolates from focus L and M, G and H possessed the same DFR profiles and similar VNTR patterns, respectively. It is also well illustrated by the bv. Medievalis isolates established with little genetic diversity from China to the Caspian Sea, and more recently by the world-wide spread of the Orientalis biovar in the course of the third pandemic.

These transmission patterns interact with each other to form the current distribution of *Y. pestis* in the world, and one pattern may take the leading role at any particular place or time. *Y. pestis* survival is the result of different adaptations to persist in the environment [3], [4], [43].

### Concluding remarks

In this study, based on 25 VNTR MLVA analysis, the data derived from more than 500 *Y. pestis* isolates were differentiated into 350 genotypes. Isolates in some foci that could not be differentiated with DFR analysis could be easily separated, illustrating the very high and meaningful resolving power of MLVA25. If needed, this resolution may be further increased by using some of the very variable loci described by Klevitska *et al*. [27]. In spite of the high homoplasy level at most VNTR loci considered individually, we observe here an excellent agreement of MLVA with other methods, including SNP, CRISPR and DFR analysis. This may be due to the use of a panel of very diverse VNTR loci, in terms of polymorphism content and consequently mutation rate and homoplasy level (Table 1). Synonymous SNP analysis together with whole genome sequencing of key strains identified here will be needed to test the proposed hypotheses: large-scale MLVA investigation suggests that only three lineages have produced the establishment of *Y. pestis* subsp. *pestis* foci outside Central Asia and China; in all three cases the limited diversity observed in these foci is compatible with clonal expansion from a unique strain; only three strains may each have caused one human pandemic; the genetic diversity observed today within the progenitors of these strains, for instance in Central Africa would be, in that case, the result of roughly 1000–2000 years of clonal expansion according to historical records [7]; this genetic divergence will have occurred independently of the Chinese lineages.

In this view, it seems very difficult to imagine that bv. Orientalis could have been the cause of all three pandemics. This would imply that, whereas bv. Orientalis very successfully led to the creation of new natural foci during the third pandemic, it failed to do so during the first two. Conversely, the role of bv. Antiqua and bv. Medievalis which similarly proved able to induce the formation of natural foci from China to Africa or to the Caspian Sea respectively are strengthened as excellent candidates for being the agent of the first and second pandemic. It is the very high typing resolution of MLVA which demonstrates the very limited genetic diversity of the *Y. pestis* subsp. *pestis* strains present outside China, compared to the extensive diversity present in China.

Data presented here will be of great assistance in the development of a genomic polymorphism database of *Y. pestis* for tracing the origin of this agent in case a plague outbreak or bioterrorism attack occurs. This method is easily standardized for data exchange, as illustrated by this study. We analyzed different *Y. pestis* isolates in laboratories in China, France and Russia, independently, without the need for strain or even DNA exchange, using a standardized protocol. The data could be exchanged and merged for combined analysis, which gave us a model how to develop databases for molecular epidemiology, surveillance or forensic microbiology of some highly dangerous pathogens.

## Supporting Information

Figure S1 The *Y. pestis* subsp. *pestis* bv. Antiqua-Medievalis group, dendrogram based on the 25 VNTR loci A color code is assigned to the main clusters: blue, predominantly bv. Antiqua from the H, KP and 38 foci, with a single strain from the G focus; green, bv. Antiqua from Tibet (G focus and Nepal); red, bv. Medievalis strains which could be subdivided into three clades: the K1 (including 8 strains from focus K1, 2 from K2 and one from focus A), J (including 9 strains from the J focus and one strain from focus I) and I foci (including 29 strains from the I focus, 5 strains from the H focus, 2 from the L, and 1 from the D focus); light blue, bv. Medievalis isolates from the Caspian Sea area, and the O focus. From left to right, strain Id, focus, genomovar.
(0.06 MB PDF)

Figure S2 The *Y. pestis* subsp. *pestis* bv. Intermedium-Antiqua-Orientalis group, Intermedium-Antiqua dendrogram based on the 25 VNTR loci The yellow color code corresponds to thirty-two “B2–B4” strains with three “K2” and one “A” exceptions; the dark green covers strains from the A-B1-33 foci, from the C focus, and from 37 with one exception coming from B2. Green colored strains contain the a37 spacer in CRISPR locus YPa [17] whereas yellow colored strains contain the a7 spacer. The grey color corresponds to bv. Antiqua strains from Africa. The light green color corresponds to bv. Antiqua strains from the C and D focus (80 strains), with two exceptions from K2. The columns from left to right indicate strain Id, focus of origin, location, host or vector, genomovar.
(0.09 MB PDF)

Figure S3 The *Y. pestis* subsp. *pestis* Orientalis group, dendrogram based on the 25 VNTR loci. The color code of the bv. Antiqua isolates from the E focus reflect the genomovar deduced from DFR analysis [28]. Some Antiqua isolates from focus E have the same genomovar as the bv. Orientalis isolates (presence of DFR13). The color of the bv. Orientalis isolates reflects the geographic origin of the isolates (country or Chinese province), except for “third pandemic” strains. Strains associated with the third pandemic are all given the same color, independently of their geographic origin. The Fujian province isolates are most closely related to the “third pandemic” group, suggesting that they represent a “third pandemic focus”, or alternatively that they are a candidate source of the third pandemic strain. In contrast, bv. Orientalis strains from the Guangxi province, Yunnan province, Vietnam, appear to be slightly more distantly related. See Figure 2 legend for columns content.
(0.09 MB PDF)

Table S1 (0.08 MB DOC)

Table S2 (0.01 MB PDF)
