# Supplementary figures and images for: Genotyping and Phylogenetic Analysis of Yersinia pestis by MLVA: Insights into the Worldwide Expansion of Central Asia Plague Foci
Source: PLoS One. 2009 Jun 22;4(6):e6000. doi: 10.1371/journal.pone.0006000 (PMC2694983; doi:10.1371/journal.pone.0006000)

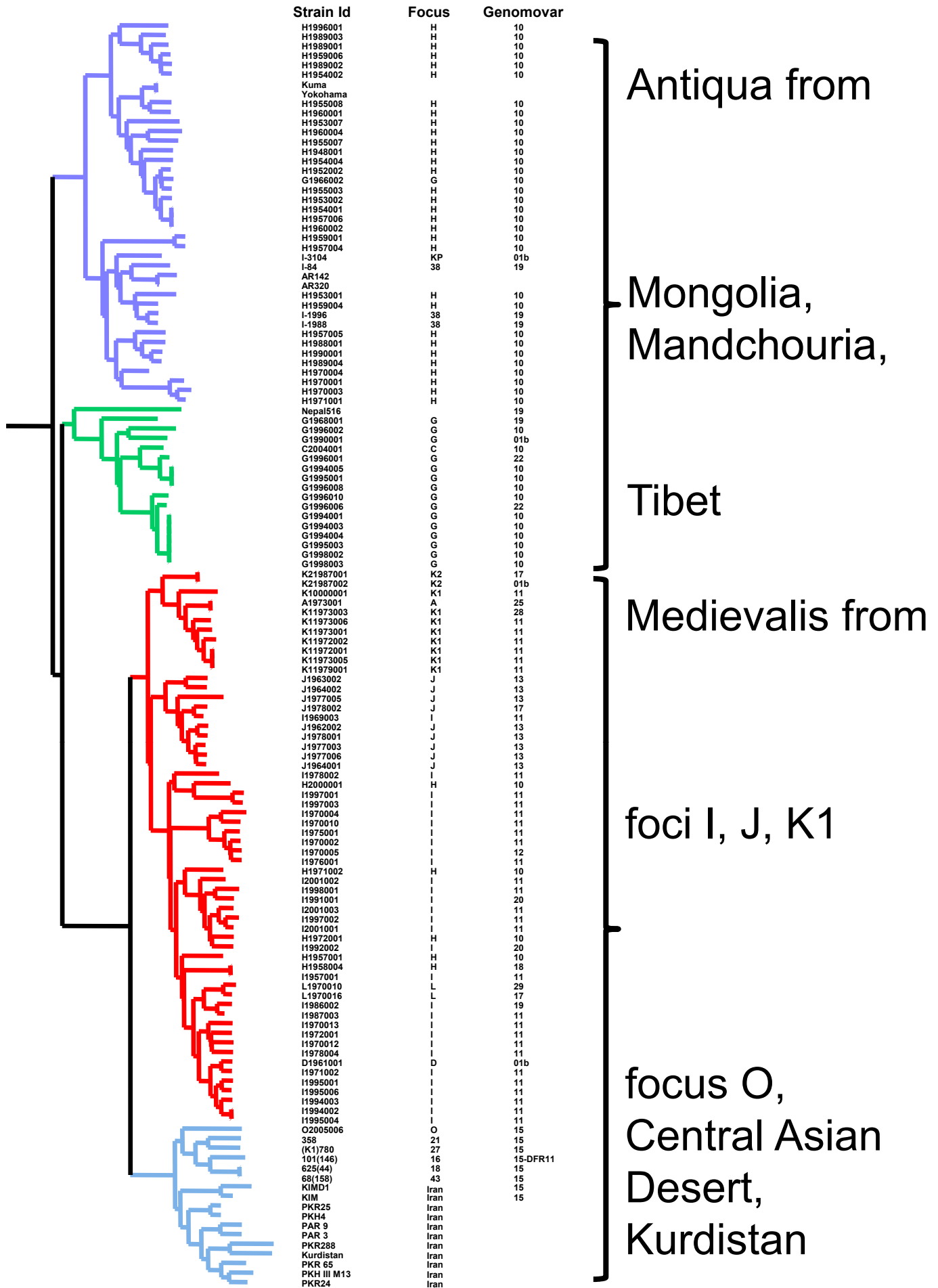

Supplement: Figure S1 — The Y. pestis subsp. pestis bv. Antiqua-Medievalis group, dendrogram based on the 25 VNTR loci A color code is assigned to the main clusters: blue, predominantly bv. Antiqua from the H, KP and 38 foci, with a single strain from the G focus; green, bv. Antiqua from Tibet (G focus and Nepal); red, bv. Medievalis strains which could be subdivided into three clades: the K1 (including 8 strains from focus K1, 2 from K2 and one from focus A), J (including 9 strains from the J focus and one strain from focus I) and I foci (including 29 strains from the I focus, 5 strains from the H focus, 2 from the L, and 1 from the D focus); light blue, bv. Medievalis isolates from the Caspian Sea area, and the O focus. From left to right, strain Id, focus, genomovar. (0.06 MB PDF) [file pone.0006000.s001.pdf]
