# Supplementary material for: Genotyping and Phylogenetic Analysis of Yersinia pestis by MLVA: Insights into the Worldwide Expansion of Central Asia Plague Foci
Source: PLoS One. 2009 Jun 22;4(6):e6000. doi: 10.1371/journal.pone.0006000 (PMC2694983; doi:10.1371/journal.pone.0006000)
Supplement: Figure S2 — The Y. pestis subsp. pestis bv. Intermedium-Antiqua-Orientalis group, Intermedium-Antiqua dendrogram based on the 25 VNTR loci The yellow color code corresponds to thirty-two “B2–B4” strains with three “K2” and one “A” exceptions; the dark green covers strains from the A-B1-33 foci, from the C focus, and from 37 with one exception coming from B2. Green colored strains contain the a37 spacer in CRISPR locus YPa [17] whereas yellow colored strains contain the a7 spacer. The grey color corresponds to bv. Antiqua strains from Africa. The light green color corresponds to bv. Antiqua strains from the C and D focus (80 strains), with two exceptions from K2. The columns from left to right indicate strain Id, focus of origin, location, host or vector, genomovar. (0.09 MB PDF) [file pone.0006000.s002.pdf]

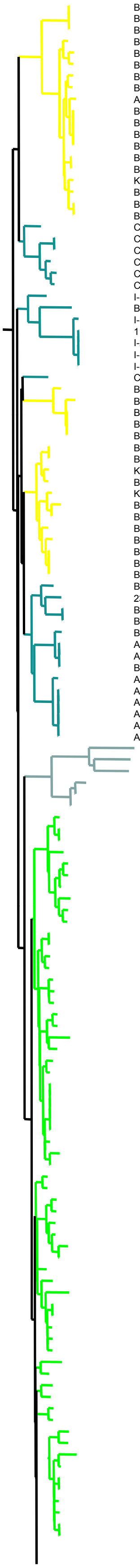

Intermedium and Antiqua from foci A, B, C, 33, 37

Strain Id	Focus	Location	Host or vector	Genomovar
B41975001	B4	Nileke, Xinjiang	*Marmota baibacina*	01a
B41976001	B4	Nileke, Xinjiang	*Spermophilus undulates*	01a
B41983001	B4	Nileke, Xinjiang	*Marmota baibacina*	01a
B21972002	B2	Hutubi,Xinjiang	patient	02
B30000013	B3	Manasi, Xinjiang	*Marmota baibacina*	02
B31971001	B3	Wusu, Xinjiang	*Marmota baibacina*	03
B31989002	B3	Wusu, Xinjiang	*Marmota baibacina*	03
B2005017	B2	Wusu, Xinjiang	*Citellophilus tesquorum*	16
A1966001	A	Wuqia,Xinjiang	*Marmota caudata*	16
B21967001	B2	Hutubi,Xinjiang	*Marmota baibacina*	no pMT
B31965001	B3	Jinghe, Xinjiang	*Spermophilus undulatus*	03
B31981001	B3	Jinghe, Xinjiang	*Spermophilus undulatus*	03
B31984001	B3	Wusu, Xinjiang	*Spermophilus undulatus*	03
B31969001	B3	Jinghe, Xinjiang	*Marmota baibacina*	03
B31982002	B3	Jinghe, Xinjiang	*Marmota baibacina*	03
K21987003	K2	Qiemo, Xinjiang	*Rhadinopsylla liventricosa*	01a
B31964002	B3	Wusu, Xinjiang	*Spermophilus undulatus*	03
B31983002	B3	Wusu, Xinjiang	*Citellophilus tesquorum*	03
B31994002	B3	Wusu, Xinjiang	*Marmota baibacina*	03
C1976008	C	Wulan,Qinghai	*Canis familiaris*	01b
C1976001	C	Akesai,Gansu	*Marmota himalayana*	01b
C1989002	C	Akesai,Gansu	*Oropsylla silantiewi*	05
C1961006	C	Akesai,Gansu	*Marmota himalayana*	01b
C1972002	C	Lenghu, Qinghai	*Marmota*	05
C1972001	C	Lenghu, Qinghai	*Oropsylla silantiewi*	01b
I-3113	37	Kyzyl-Khaya Tract, right bank of the Barlyk river, Sagly mesofocus, Tuva focus	*Citellus undulatus*	01b
B2003030	B2	Wenquan, Xinjiang	*Marmota baibacina*	01a
I-3450	37	Kadyr-Oruk Tract, Mongun-Taigin mesofocus, Tuva focus	*Citellus undulatus*	02-DFR23
1192	37	Tuva focus, Russia	*Citellus undulatus*	02-DFR23
I-2638	37	Yigylak Tract, Mongun-Taigin mesofocus, Tuva focus, Russia	*Citellus undulatus*	02-DFR23
I-3448	37	Buure Tract, Mongun-Taigin mesofocus, Tuva focus, Russia	*Citellus undulatus*	02-DFR23
I-3449	37	Buure Tract, Mongun-Taigin mesofocus, Tuva focus, Russia	*Citellus undulatus*	02-DFR23
C2004007	C	Qinghai	patient	01b
B31978001	B3	Wusu, Xinjiang	*Marmota baibacina*	02
B32002002	B3	Wusu, Xinjiang	*Marmota baibacina*	31
B31988004	B3	Shawan, Xinjiang	patient	31
B31994001	B3	Wusu, Xinjiang	*Marmota baibacina*	02
B31994003	B3	Shawan, Xinjiang	*Pulex irritans*	no pMT
B21983001	B2	Changji, Xinjiang	*Marmota baibacina*	02
B21984003	B2	Changji, Xinjiang	*Neoheomatopinus plaearctus*	02
K21985005	K2	Ruoqiang,Xinjiang	*Oropsylla silantiewi*	31
B21980001	B2	Hutubi,Xinjiang	*Oropsylla silantiewi*	02
K21985001	K2	Ruoqiang,Xinjiang	*Oropsylla silantiewi*	31
B31982001	B3	Jinghe, Xinjiang	*Marmota baibacina*	no pMT
B31972001	B3	Jinghe, Xinjiang	*Spermophilus undulatus*	02
B21959001	B2	Manasi, Xinjiang	*Marmota baibacina*	02
B21989001	B2	Changji, Xinjiang	*Marmota baibacina*	02
B21959002	B2	Hutubi,Xinjiang	*Marmota baibacina*	02
B21965001	B2	Manasi, Xinjiang	*Marmota baibacina*	02
B31967002	B3	Wusu, Xinjiang	*Marmota baibacina*	02
B11992002	B1	Atushi, Xinjiang	*Marmota baibacina*	04
231	33	Aksai focus, Kirghizia	*Marmota baibacina*	04-DFR18
B11979001	B1	Atushi, Xinjiang	*Marmota baibacina*	04
B11995002	B1	Atushi, Xinjiang	*Marmota baibacina*	04
B12001001	B1	Wuqia,Xinjiang	*Marmota baibacina*	01b
A1992001	A	Wuqia,Xinjiang	*Oropsylla silantiewi*	04
A1996002	A	Wuqia,Xinjiang	*Capra ibex*	04
B11989001	B1	Wuqia,Xinjiang	*Marmota baibacina*	04
A1956001	A	Wuqia,Xinjiang	*Marmota caudata*	04
A1983001	A	Wuqia,Xinjiang	*Marmota caudata*	04
A1995001	A	Wuqia,Xinjiang	*Marmota caudata*	04
A1996001	A	Wuqia,Xinjiang	*Marmota caudata*	04
A1997001	A	Wuqia,Xinjiang	*Marmota caudata*	04
A1998002	A	Wuqia,Xinjiang	*Marmota caudata*	04

Antiqua from Africa

Strain Id	Focus	Location	Host or vector	Genomovar
Margaret Muga		Kenya	patient	
343 A		RDC (Belgian Congo)		
Antiqua		RDC (Belgian Congo)	no data	05
129 M22		Kenya		
Lita 543		RDC (Belgian Congo)		
147 537		Kenya		

Antiqua from foci C and D

Strain Id	Focus	Location	Host or vector	Genomovar
D1982001	D	Sunan,Gansu	patient	08
C1954002	C	Henan,Qinghai	*Ochotona daurica*	05
D1985001	D	Sunan,Gansu	*Marmota himalayana*	08
D1984001	D	Sunan,Gansu	*Callopsylla dolabris*	08
D1958001	D	Qilian,Qinghai	*Marmota himalayana*	05
D1964002	D	Menyuan,Qinghai	*Canis familiaris*	08
D1965001	D	Qilian,Qinghai	*Mustela eversmanni*	08
D1964001	D	Menyuan, Qinghai	*Marmota*	05
D0000004	D	Menyuan,Qinghai		08
D1977001	D	Qilian,Qinghai	*Marmota himalayana*	08
C1967002	C	Gonghe,Qinghai	patient	08
C1970005	C	Gonghe,Qinghai	patient	08
D1963002	D	Menyuan,Qinghai	*Mus musculus*	08
C1967003	C	Gonghe,Qinghai	*Pulex irritans*	08
C1971001	C	Huangyuan, Qinghai	patient	07
C1967001	C	Wulan,Qinghai	*Marmota*	08
C1972004	C	Yumen, Gansu	patient body	08
D0000003	D	Qilian,Qinghai		08
D0000002	D	Qilian,Qinghai		08
C2004005	C	Qinghai	patient	08
C1988001	C	Xinghai,Qinghai	*Linognathoides palaearctus*	08
C1977001	C	Yumen, Gansu	patient	08
C1964003	C	Haiyan,Qinghai	*Ixodes crenulatus*	08
C1961002	C	Gonghe,Qinghai	*Callopsylla dolabris*	08
C1964006	C	Haiyan,Qinghai	*Oropsylla silantiewi*	08
C1974001	C	Gangcha,Qinghai	*Rhadinopsylla liventricosa*	08
C1979002	C	Mangya,Qinghai	*Marmota himalayana*	08
D0000001	D	Qilian,Qinghai		08
C1961004	C	Sunan,Gansu	*Marmota himalayana*	08
C0000002	C	Gonghe,Qinghai	*Pulex irritans*	08
D1963003	D	Menyuan,Qinghai	*Neopsylla hongyangensis*	08
D2001002	D	Tongde,Qinghai	patient	08
D2001001	D	Tongde,Qinghai	*Vulpes vulpes*	08
D1991003	D	Tongde,Qinghai	*Marmota himalayana*	08
D1991005	D	Zeku,Qinghai	*Allactaga sibirica*	08
D1991002	D	Zeku,Qinghai	*Marmota himalayana*	08
D1991006	D	Zeku,Qinghai	*Frontopsylla wagneri*	08
C1997001	C	Angqian, Qinghai	*Ovis aries*	07
D1991004	D	Zeku,Qinghai	*Marmota*	01b
C1959001	C	Xiahe,Gansu	*Marmota himalayana*	08
C1986001	C	Tongren, Qinghai	*Marmota*	07
C2000001	C	Delingha, Qinghai	*Neoheomatopinus palaearctus*	07
C1960001	C	Tongren, Qinghai	patient	07
C1964004	C	Gannan, Gansu	*Marmota himalayana*	07
C0000001	C	Xunhua, Qinghai	*Marmota*	07
C1960003	C	Xiahe, Gansu	*Marmota himalayana*	07
C1960004	C	Xiahe, Gansu	*Marmota himalayana*	07
C1970009	C	Xunhua, Qinghai	*Marmota*	07
C1978001	C	Maduo, Qinghai	*Marmota*	01b
C1954001	C	Guinan,Qinghai	*Marmota himalayana*	05
C1969002	C	Wulan,Qinghai	*Rhipicephalus sanguineus*	08
K21985008	K2	Ruoqiang,Xinjiang	*Marmota himalayana*	05
K21985002	K2	Ruoqiang,Xinjiang	*Marmota himalayana*	05
C1978009	C	Dingqing,Tibet	*Marmota himalayana*	05
C1989001	C	Zhaduo,Qinghai	patient	01b
C1978008	C	Zhiduo,Qinghai	*Felis sp*	05
C1992001	C	Nierong,Tibet	*Marmota himalayana*	05
C1976002	C	Nagqu,Tibet	*Marmota Himalayana*	06
C1990003	C	Anduo,Tibet	*Marmota himalayana*	05
C1985003	C	Guinan,Qinghai	*Marmota himalayana*	05
C1994002	C	Biru ,Tibet	*Ovis aries*	06
C1970004	C	Qumalai,Qinghai	*Marmota himalayana*	05
