# Supplementary material for: Genotyping and Phylogenetic Analysis of Yersinia pestis by MLVA: Insights into the Worldwide Expansion of Central Asia Plague Foci
Source: PLoS One. 2009 Jun 22;4(6):e6000. doi: 10.1371/journal.pone.0006000 (PMC2694983; doi:10.1371/journal.pone.0006000)
Supplement: Figure S3 — The Y. pestis subsp. pestis Orientalis group, dendrogram based on the 25 VNTR loci. The color code of the bv. Antiqua isolates from the E focus reflect the genomovar deduced from DFR analysis [28]. Some Antiqua isolates from focus E have the same genomovar as the bv. Orientalis isolates (presence of DFR13). The color of the bv. Orientalis isolates reflects the geographic origin of the isolates (country or Chinese province), except for “third pandemic” strains. Strains associated with the third pandemic are all given the same color, independently of their geographic origin. The Fujian province isolates are most closely related to the “third pandemic” group, suggesting that they represent a “third pandemic focus”, or alternatively that they are a candidate source of the third pandemic strain. In contrast, bv. Orientalis strains from the Guangxi province, Yunnan province, Vietnam, appear to be slightly more distantly related. See Figure 2 legend for columns content. (0.09 MB PDF) [file pone.0006000.s003.pdf]

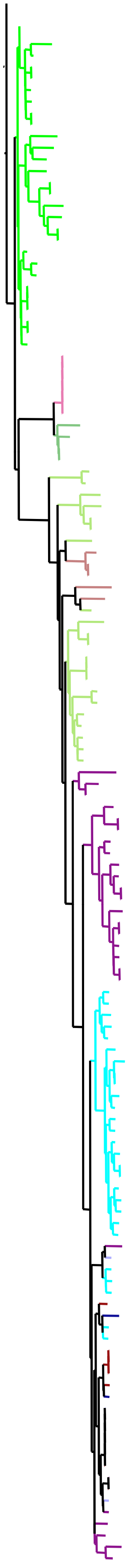

Antiqua focus C (continued)

Strain Id	Focus	bv.	Geographic origin	Host or vector	Genomovar
C1992001	C	Antiqua	Nierong,Tibet	*Marmota himalayana*	05
C1976002	C	Antiqua	Nagqu,Tibet	*Marmota Himalayana*	06
C1990003	C	Antiqua	Anduo,Tibet	*Marmota himalayana*	05
C1985003	C	Antiqua	Guinan,Qinghai	*Marmota himalayana*	05
C1994002	C	Antiqua	Biru ,Tibet	*Ovis aries*	06
C1970004	C	Antiqua	Qumalai,Qinghai	*Marmota himalayana*	05
C1988004	C	Antiqua	Nierong,Tibet	patient	05
C1964007	C	Antiqua	Yushu,Qinghai	*Marmota himalayana*	05
C1975003	C	Antiqua	Yushu,Qinghai	*Ovis aries*	05
C1990002	C	Antiqua	Baqing,Tibet	*Marmota himalayana*	05
C1974005	C	Antiqua	Zhaduo,Qinghai	*Felis sp*	05
C1962003	C	Antiqua	Xinghai,Qinghai	*Pulex irritans*	05
C1964005	C	Antiqua	Gonghe,Qinghai	patient	05
C1999001	C	Antiqua	Shiqu, Sichuan	patient	
C1990004	C	Antiqua	Angqian, Qinghai	*Marmota*	01b
C1978003	C	Antiqua	Yushu,Qinghai	*Procapra picticaudata*	05
C1978011	C	Antiqua	Angqian, Qinghai	*Meles meles*	05
C1983002	C	Antiqua	Changdu,Tibet	*Marmota himalayana*	05
C1978013	C	Antiqua	Gomud,Qinghai	*Lynx lynx*	05
C1991001	C	Antiqua	Gomud,Qinghai	*Marmota himalayana*	05
C1978002	C	Antiqua	Naqu, Tibet	*Marmota himalayana*	06
C1980002	C	Antiqua	Qumalai,Qinghai	patient	05
C1983004	C	Antiqua	Chaya,Tibet	*Marmota himalayana*	05
C1976003	C	Antiqua	Naqu, Tibet	*Marmota himalayana*	06
C1978004	C	Antiqua	Naqu, Tibet	*Marmota himalayana*	06
C1976007	C	Antiqua	Naqu, Tibet	*Marmota himalayana*	06

Antiqua focus E

Strain Id	Focus	bv.	Geographic origin	Host or vector	Genomovar
E1954001	E	Antiqua	Jianchuan, Yunnan	*Eothenomys miletus*	07
E1977001	E	Antiqua	Jianchuan, Yunnan	*Ctenophthalmus quadratus*	07
E1979002	E	Antiqua	Jianchuan, Yunnan	*Stenischia humilis*	07
E1982001	E	Antiqua	Jianchuan, Yunnan	*Apodemus chevrieri*	07
E1983004	E	Antiqua	Jianchuan, Yunnan	*Eothenomys miletus*	07
E1984002	E	Antiqua	Jianchuan, Yunnan	*Neopsylla specialis Jordan*	07
E1977003	E	Antiqua	Jianchuan, Yunnan	*Apodemus speciosus*	09
F1954001	F	Antiqua	Xiangyun, Yunnan	*Rattus flavipectus*	09
E1954003	E	Antiqua	Jianchuan, Yunnan	*Stenischia humilis*	09
E1957001	E	Antiqua	Jianchuan, Yunnan	*Neopsyllini specialis*	09

Orientalis, Fujian and “Third pandemic”

Strain Id	Focus	bv.	Geographic origin	Host or vector	Genomovar
Java9		Orientalis	Surakarta, Java, Indonesia		
Java10		Orientalis	Surakarta, Java, Indonesia	patient	
Johannesburg K120-043		Orientalis	South Africa	patient	
195/P		Orientalis	India		
M23		Orientalis			
GB		Orientalis	UK		
Fay 524		Orientalis	Senegal		
F1943001	F	Orientalis	Fuzhou, Fujian	patient	09
F1950001	F	Orientalis	Hua'an, Fujian	*Rattus*	09
F1952004	F	Orientalis	Putian, Fujian	*Rattus*	09
F1946001	F	Orientalis	Gutian, Fujian	patient	09
F1943002	F	Orientalis	Fuzhou, Fujian	patient	09
CO92		Orientalis	Colorado, USA	patient	09
Thierno 523		Orientalis	Senegal		
Exu38		Orientalis	Exu, Pernambuco, Brasil		
Exu21		Orientalis	Exu, Pernambuco, Brasil		
TS		Orientalis			
TW		Orientalis			
Yp1255		Orientalis			
Barabanki 4		Orientalis	Barabanki, Uttar Pradesh, India		
Barabanki 3		Orientalis	Barabanki, Uttar Pradesh, India		
IP 16=6/69		Orientalis	Madagascar		
Hambourg9 695		Orientalis	Germany		
MAKAN 020		Orientalis	Senegal	patient	
EV76		Orientalis	Madagascar	patient	09-DFR10
G32pgm+		Orientalis			

Orientalis from Yunnan, Guangxi provinces

and other countries (Vietnam, Myanmar)

Strain Id	Focus	bv.	Geographic origin	Host or vector	Genomovar
E1954002	E	Orientalis	Jianchuan, Yunnan	*Rattus flavipectus*	09
E1984001	E	Orientalis	Jianchuan, Yunnan	*Apodemus chevrieri*	09
E1984005	E	Orientalis	Jianchuan, Yunnan	*Rattus nitidus*	09
F1953002	F	Orientalis	Baoshan, Yunnan	*Rattus flavipectus*	07
F1953003	F	Orientalis	Midu, Yunnan	*Rattus flavipectus*	09
F1955001	F	Orientalis	Xiangyun, Yunnan	*Rattus flavipectus*	09
F1990013	F	Orientalis	Yingjiang, Yunnan	patient	09
F1990015	F	Orientalis	Lianghe, Yunnan	*Rattus flavipectus*	09
F1990010	F	Orientalis	Longchuan, Yunnan	*Xenopsylla cheopis*	09
F1982002	F	Orientalis	Longchuan, Yunnan	*Rattus flavipectus*	09
F1991002	F	Orientalis	Wanding, Yunnan	*Rattus flavipectus*	09
F1996007	F	Orientalis	Mojiang, Yunnan	*Xenopsylla cheopis*	09
F1984002	F	Orientalis	Yingjiang, Yunnan	*Rattus flavipectus*	09
F1991011	F	Orientalis	Gengma, Yunnan	*Rattus flavipectus*	09
F1992001	F	Orientalis	Lancang, Yunnan	*Rattus flavipectus*	09
F1984003	F	Orientalis	Yingjiang, Yunnan	*Xenopsylla cheopis*	09
F1983006	F	Orientalis	Baoshan, Yunnan	*Leptopsylla segnis*	09
F1982003	F	Orientalis	Ruili, Yunnan	*Rattus flavipectus*	09
F1992012	F	Orientalis	Cangyuan, Yunnan	*Rattus flavipectus*	09
Dalat 129	Viet.	Orientalis	Dalat, Vietnam	patient	
Dalat 161	Viet.	Orientalis	Dalat, Vietnam	patient	
Dalat 286-06	Viet.	Orientalis	Dalat, Vietnam	patient	
Dalat 266	Viet.	Orientalis	Dalat, Vietnam	patient	
Dalat 268	Viet.	Orientalis	Dalat, Vietnam	patient	
Dalat 320	Viet.	Orientalis	Dalat, Vietnam	patient	
Saigon 64.1244	Viet.	Orientalis	Saigon, Vietnam	patient	
Saigon 64.1265	Viet.	Orientalis	Saigon, Vietnam	patient	
Saigon 64.1264	Viet.	Orientalis	Saigon, Vietnam	patient	
Saigon 64.1318	Viet.	Orientalis	Saigon, Vietnam	patient	
Dalat 245	Viet.	Orientalis	Dalat, Vietnam	patient	
Dalat 269	Viet.	Orientalis	Dalat, Vietnam	patient	
Dalat 146	Viet.	Orientalis	Dalat, Vietnam	patient	
Saigon 64.1205	Viet.	Orientalis	Saigon, Vietnam	patient	
Dalat 272	Viet.	Orientalis	Dalat, Vietnam	patient	
Dalat	Viet.	Orientalis	Dalat, Vietnam	patient	
Dalat 270	Viet.	Orientalis	Dalat, Vietnam	patient	
Dalat 267	Viet.	Orientalis	Dalat, Vietnam	patient	
Dalat 260	Viet.	Orientalis	Dalat, Vietnam	patient	
Dalat 180	Viet.	Orientalis	Dalat, Vietnam	patient	
Dalat 321	Viet.	Orientalis	Dalat, Vietnam	patient	
Dalat 318	Viet.	Orientalis	Dalat, Vietnam	patient	
F1984001	F	Orientalis	Yingjiang, Yunnan	*Rattus flavipectus*	18
F2002007	F	Orientalis	Xingyi, Guizhou	*Rattus flavipectus*	09
Dalat 299	Viet.	Orientalis	Dalat, Vietnam	patient	
Dalat 256	Viet.	Orientalis	Dalat, Vietnam	patient	
Dalat 278	Viet.	Orientalis	Dalat, Vietnam	patient	
F2000008	F	Orientalis	Longlin, Guangxi	patient	09
F1991009	F	Orientalis	Courageous, Myanmar	*Rattus flavipectus*	09
Dalat 296	Viet.	Orientalis	Dalat, Vietnam	patient	
Dalat 292	Viet.	Orientalis	Dalat, Vietnam	patient	
F0000002	F	Orientalis	Longlin, Guangxi		09
F1991003	F	Orientalis	Wanding, Yunnan	*Rattus flavipectus*	09
F2000006	F	Orientalis	Longlin, Guangxi	*Rattus flavipectus*	09
F1982008	F	Orientalis	Longchuan, Yunnan	*Xenopsylla cheopis*	09
F1995007	F	Orientalis	Myanmar	*Rattus flavipectus*	09
F2000002	F	Orientalis	Xingyi, Guizhou	*Rattus flavipectus*	09
F2000009	F	Orientalis	Longlin, Guangxi	patient	09
F2002006	F	Orientalis	Xingyi, Guizhou	*Rattus flavipectus*	09
Birmanie 637	Myan.	Orientalis	Myanmar		
Birmanie 548	Myan.	Orientalis	Myanmar		
F2000010	F	Orientalis	Longlin, Guangxi	*Rattus flavipectus*	09
F1993001	F	Orientalis	Lancang, Yunnan	*Rattus flavipectus*	18
F2000007	F	Orientalis	Longlin, Guangxi	*Rattus flavipectus*	09
F2000003	F	Orientalis	Xingyi, Guizhou	*Rattus flavipectus*	09
F1996001	F	Orientalis	Mojiang, Yunnan	*Rattus flavipectus*	09
F1996002	F	Orientalis	Mojiang, Yunnan	*Rattus flavipectus*	18
F2002001	F	Orientalis	Honghe, Yunnan	*Rattus flavipectus*	09
F1991007	F	Orientalis	Courageous, Myanmar	*Rattus flavipectus*	09
F1991006	F	Orientalis	Zhenkang, Yunnan	*Rattus flavipectus*	09
