# Supplementary material for: Genotyping and Phylogenetic Analysis of Yersinia pestis by MLVA: Insights into the Worldwide Expansion of Central Asia Plague Foci
Source: PLoS One. 2009 Jun 22;4(6):e6000. doi: 10.1371/journal.pone.0006000 (PMC2694983; doi:10.1371/journal.pone.0006000)
Supplement: Table S1 — (0.08 MB DOC) [file pone.0006000.s004.doc]

## *Supplementary Table 1. Taxonomic characters of strains, which distinguish different Y. pestis subspecies and compliance of subspecies with biovars a*

| **Biovar** | **Subspecies** | **Fermentation of** | | | | | **Nitrate reduction** | **urease activity** | **Pesticin I production** | **Susceptibility to pesticin I** | **Fibrinolytic activity** | **Coagulase activity** | **Dependency upon nutrition factors** | | | | | | | | **Virulence for guinea pigs** | **Region of circulation** | Hosts | **Mutation rate of Pgm^+^ to Pgm^–^ in 10 generations (%)** |
| --- | --- | --- | --- | --- | --- | --- | --- | --- | --- | --- | --- | --- | --- | --- | --- | --- | --- | --- | --- | --- | --- | --- | --- | --- |
|  |  | **rhamnose** | **melibiose** | **arabinose** | **glycerol** | **melezitose** |  |  |  |  |  |  | **leucine** | **methionine** | **arginine** | **thiamine** | **cysteine** | **phenylalanine** | **threonine** | **tyrosine** |  |  |  |  |
| Microtus | *angola ^b^* | **+** *^c^* | **?***^d^* | **+** *^e^* | **+***^f^* | **?** | **–** *^g^* | **?** | **?** | **?** | **?** | **?** | **?** | **?** | **?** | **?** | **?** | **?** | **?** | **?** | **–** | Angola | **?** | **0** |
| Microtus | *caucasica ^h^* | **+** | **+** | **+** | **+** | **–** | **+** | **–** | **–** | **+** | **–** | **–** | **±** | **+** | **+** | **+** | **±** | **+** | **+** | ? | **–** | Transcaucasian highland, Armenia, Azerbaijan and Georgia; Mountain Dagestan, Russia | *Microtus arvalis* | **0** |
| Microtus | *ulegeica* | **+** | **+** | **+** | **+** | ? | **–** | **–** | **+** | **+/–***^g^* | **+** | **+** | **–** | **–** | **–** | **–** | **+** | **+** | ? | ? | **–** | Gobi Desert, Northeast Mongolia | *Microtus gregalis, Alticola strelzovi, Ochotona pallasi pricei* | **0** |
| Microtus | *altaica* | **+** | **+** | **–** | **+** | ? | **–** | **–** | **+** | **+** | **+** | **+** | **+** | **–** | **+** | **–** | **+** | **+** | ? | ? | **–** | Mountain Altai, Russia and Mongolia | *Microtus gregalis, Alticola strelzovi, Ochotona pallasi pricei* | **0** |
| Microtus | *hissarica* | **+** | **+** | **–** | **+** | **±** | **–** | **±** | **+** | **±** | **+** | **+** | **+** | **+** | **–** | **–** | **+** | **+** | **–** | **–** | **–** | Hissarian ridge, Tadjikistan and Uzbekistan | *Microtus carruthersi* | **0** |
| Microtus | *xilingolensis* | **+** | **+** | **–** | **+** | **?** | **–** | **–** | **+** | **+** | **?** | **?** | **–** | **–** | **–** | **?** | **–** | **–** | **?** | **?** | **–** | Xilin Gol Grassland, Inner Mogolia, China | *Microtus brandti* | **0** |
| Microtus | *qinghaiensis* | **+** | **+** | **–** | **+** | **–** | **–** | **–** | **+** | **?** | **?** | **?** | **–** | **–** | **–** | **?** | **–** | **–** | **?** | **?** | **–** | Qinghai–Tibet Plateau, Qinghai and Sichuan, China | *Microtus fuscus* | **0** |
| Microtus | *talassica* | **+** | **+** | **–** | **+** | **–** | **–** | **+** | **+** | **–** | **+** | **+** | **+** | ? | **+** | ? | **+** | **+** | ? | **+** | **–** | Talassian ridge, Kirghizia | *Microtus gregalis, Marmota caudata* | **0** |
| Intermedium | *pestis* | **+** | **+** | **+** | **+** | **?** | **+** | **?** | **+** | **?** | **?** | **?** | **-** | **+** | **-** | **?** | **+** | **±** | **±** | **?** | **+** | North Tianshan, Xinjiang, China | *Marmota baibacina, Spermophilus undulatus* | **0** |
| Antiqua | *pestis* | **–** | **–** | **+** | **+** | **–** | **+** | **–** | **+** | **–** | **+** | **+** | **±** | **+** | **–** | **–** | **±** | **±** | **+** | **–** | **+** | Central and northern Asia, central Africa | *Marmota* spp. | **> 20** |
| Medievali*s* | *pestis* | **–** | **±** | **+** | **+** | **–** | **–** | **–** | **+** | **–** | **+** | **+** | **±** | **+** | **–** | **–** | **±** | **±** | **+** | **–** | **+** | Central Asia | *Citellus* (*Spermophilus*) spp.*, Meriones* spp., *Rhombomys* spp. | **> 20** |
| Orientalis | *pestis* | **–** | **–** | **+** | **–** | **–** | **+** | **±** | **+** | **–** | **+** | **+** | **±** | **+** | **–** | **–** | **±** | **±** | **+** | **–** | **+** | Global distribution | *Rattus* spp.*,* *Cynomys* spp.*, Spermophilus* spp., *Cavia* spp., *Peromyscus* spp., *etc*. | **> 20** |

*^a^* This table was compiled from references (**Anisimov, A. P., L. E. Lindler, and G. B. Pier.** 2004. Intraspecific diversity of *Yersinia pestis*. Clin Microbiol Rev **17:**434-64; **Zhou, D., Y. Han, Y. Song, P. Huang, and R. Yang.** 2004. Comparative and evolutionary genomics of *Yersinia pestis*. Microbes Infect **6:**1226-34; **Cui, Y., Y. Li, O. Gorge, M. E. Platonov, Y. Yan, Z. Guo, C. Pourcel, S. V. Dentovskaya, S. V. Balakhonov, X. Wang, Y. Song, A. P. Anisimov, G. Vergnaud, and R. Yang.** 2008. Insight into microevolution of *Yersinia pestis* by clustered regularly interspaced short palindromic repeats. PLoS ONE **3:**e2652) and from the table proposed by the conference of experts of the Anti-Plague Establishments of the Soviet Union (Saratov, 1985) for the plague pathogen that were isolated from the territory of the FSU and Mongolia.

*^b^* The characters of Angola are similar to that of Microtus biovar “Medievalis”-like (glycerol positive, arabinose positive, and nitrate negative) (**Dongsheng Zhou, Zongzhong Tong, Yajun Song, Yanping Han**. 2004. Genetics of metabolic variations between *Yersinia pestis* biovars and the proposal of a new biovar, Microtus. J. Bacteriol. **186**: 5147–5152).

*^c^*.+, presence of a character; **–**, absence of a character; **±,** presence of a character but not in all strains.

*^d^*. No data.

*^e^* No 122 bp-deletion is detected in *araC* of Angola. This deletion may account for the inactivation of the *ara* operon involved in arabinose utilization in 91001.

*^f^* Intact *glpD* gene in Angola. 93 bp loss may result in glycerol-negative phenotype of CO92.

*^g^* A point mutation at the 1021^th^ site within *napA* gene (C->T), most likely results in the nitrate-negative phenotype of Angola.

*^h^* strains *caucasica* are deficient in pPst plasmid.

*^g^* Strains are susceptible to pesticin of strains from subspecies *pestis* and *altaica*, and also resistant to pesticin from strains of its own subspecies.
