# Supplementary material for: Genotyping and Phylogenetic Analysis of Yersinia pestis by MLVA: Insights into the Worldwide Expansion of Central Asia Plague Foci
Source: PLoS One. 2009 Jun 22;4(6):e6000. doi: 10.1371/journal.pone.0006000 (PMC2694983; doi:10.1371/journal.pone.0006000)
Supplement: Table S2 — (0.01 MB PDF) [file pone.0006000.s005.pdf]

Supplementary Table 2 List of the isolates used for genotyping

Focus	Number of isolates	Biovar	Geographical origin
A	10	Antiqua	Xinjiang, China
A	1	Medievalis	Xinjiang, China
B1	5	Antiqua	Xinjiang, China
B1	1	Medievalis	Xinjiang, China
B2	9	Antiqua	Xinjiang, China
B3	20	Antiqua	Xinjiang, China
B4	7	Antiqua	Xinjiang, China
C	54	Antiqua	Qinghai, China
C	17	Antiqua	Gansu, China
C	10	Antiqua	Tibet, China
D	20	Antiqua	Qinghai, China
D	3	Antiqua	Gansu, China
E	13	Antiqua	Yunnan, China
E	3	Orientalis	Yunnan, China
F	1	Antiqua	Yunnan, China
F	24	Orientalis	Yunnan, China
F	7	Orientalis	Guangxi, China
F	6	Orientalis	Guizhou, China
F	5	Orientalis	Fujian, China
F	4	Orientalis	Myanmar
G	16	Antiqua	Tibet, China
H	1	Ypseu**	Jilin, China
H	9	Antiqua	Jilin, China
H	21	Antiqua	Inner Mongolia, China
H	1	Antiqua	Heilongjiang, China
H	4	Medievalis	Inner Mongolia, China
H	1	Medievalis	Jilin, China
I	14	Medievalis	Inner Mongolia, China
I	6	Medievalis	Ningxia, China
I	9	Medievalis	Hebei, China
I	4	Medievalis	Shanxi, China
J	5	Medievalis	Gansu, China
J	7	Medievalis	Ningxia, China
K1	11	Medievalis	Xinjiang, China
K2	9	Antiqua	Xinjiang, China
L	10	Microtus/*xilingolensis*	Inner Mongolia, China
L	2	Medievalis	Inner Mongolia, China
M	2	Antiqua	Qinghai, China
M	20	Microtus/*qinghaiensis*	Sichuan, China
M	11	Microtus/*qinghaiensis*	Qinghai, China
O	1	Medievalis	Xinjiang, China
36*	9	Microtus/*altaica*	Mountain-Altai focus, Russia

BP*	3	Microtus/*ulegeica*	Bayanölgie province (aymag), Mongolia
4-6*	3	Microtus/*caucasica*	Tran-Caucasian-highland focus, Armenia
5 or 6*	2	Microtus/*caucasica*	Azerbaijan, Tran-Caucasian-highland focus
34*	2	Microtus/*hissarica*	Gissar focus, Tadjikistan
33*	1	Antiqua	Aksai focus, Kirghizia
MO*	1	Antiqua	Khentei, Mongolia
37*	6	Antiqua	Tuva focus, Russia
38*	3	Antiqua	Trans-Baikal focus, Russia
27*	1	Medievalis	Kyzyl-Kum focus, Uzbekistan, Kazakhstan
21*	1	Medievalis	North-Pre-Aral focus, Kazakhstan
18*	1	Medievalis	Ural-Emba focus, Kazakhstan
16*	1	Medievalis	Volga-Ural sandy focus, Russia
43*	1	Medievalis	Pre-Caspian sandy focus, Russia
/	1	Medievalis	KIMD1 (Iran/Kurdistan) used for study in lab
/	1	Antiqua	From human laboratory-acquired infection case, FSU
/	1	Orientalis	EV76 (Madagascar), live attenuated vaccine strain

* The foci in the Central Asia.

** *Y. pseudotuberculosis*
